# HIV pre-exposure prophylaxis programmatic preferences among people who inject drugs: findings from a discrete choice experiment

**DOI:** 10.1186/s13722-024-00505-2

**Published:** 2024-11-12

**Authors:** William H. Eger, Angela R. Bazzi, Chad J. Valasek, Carlos F. Vera, Alicia Harvey-Vera, Steffanie A. Strathdee, Heather A. Pines

**Affiliations:** 1 School of Medicine, University of California, San Diego, La Jolla, San Diego, CA USA; 2 School of Social Work, San Diego State University, San Diego, CA USA; 3 Herbert Wertheim School of Public Health and Human Longevity Science, University of California, San Diego, La Jolla, San Diego, CA USA; 4 Boston University School of Public Health, Boston, MA USA; 5 Division of Epidemiology and Biostatistics, School of Public Health, San Diego State University, San Diego, USA

**Keywords:** Pre-exposure prophylaxis, Substance abuse, Intravenous, Community health services, Prescriptions, Telemedicine, Discrete choice experiment

## Abstract

**Background:**

Pre-exposure prophylaxis (PrEP) holds promise for decreasing new HIV infections among people who inject drugs (PWID), yet daily oral PrEP use is low, and PrEP modality and delivery strategy preferences in this population remain understudied.

**Methods:**

From May 2022-June 2023, we conducted a discrete choice experiment (DCE) with PWID in San Diego, California. Participants viewed 18 PrEP program scenarios in sets of three and chose their preferred scenario within each set. Scenarios consisted of various combinations of five characteristics: PrEP modality (injectable, implantable, oral), frequency of use (annual, bi-monthly, daily), service location (community-based organization, clinic, telemedicine), prescription access location (on-site, street outreach, mail), and adherence supports (social support, outreach worker, phone/text reminder). Multinomial logit regression estimated probabilities of choosing PrEP program scenarios as a function of the five characteristics to estimate part-worth utility scores (PWUS; reflecting relative preferences for specific characteristic values) and relative importance scores (RIS; reflecting the relative influence of each characteristic on program choice). We also explored differences by hypothesized modifiers of preferences (i.e., sex assigned at birth, housing status, injection frequency, prior PrEP awareness).

**Results:**

Among 262 participants, mean age was 43.1 years, and most reported male sex assigned at birth (69.5%), identified as non-Hispanic (60.3%), and were previously unaware of PrEP (75.2%). Frequency of use (RIS: 51.5) and PrEP modality (RIS: 35.3) had the greatest influence on PrEP program choice. Within these characteristics, participants had relative preferences for annual use (PWUS: 0.83) and oral PrEP (PWUS: 0.57), and relative aversions to daily use (PWUS: -0.76) and implantable PrEP (PWUS: -0.53). Generally, participants did not indicate preferences for specific service or prescription access locations, or adherence supports; however, among those with prior PrEP awareness, prescription access location and adherence supports had a slightly greater influence on PrEP program choices.

**Conclusion:**

Our study considered diverse PrEP scenarios and highlighted potential preferences for long-acting oral modalities. Although not currently available, renewed investment in long-acting oral PrEP formulations may facilitate PrEP care engagement among PWID. Additional delivery and implementation strategy research is needed to support PrEP uptake and persistence in this population.

## Introduction

People who inject drugs (PWID) account for nearly 1 in 15 HIV infections in the United States and are considered a key population for efforts to end the HIV epidemic globally [1–3]. Despite a high burden of sexual and injection-related HIV exposures, and many social and structural vulnerabilities (e.g., stigma, homelessness, criminalization of drug use) that also increase risk for HIV [4, 5], the use of pre-exposure prophylaxis (PrEP) remains low in this population [6, 7]. For example, as few as one in five PWID across diverse settings have ever heard of oral PrEP, and less than 1% have ever used it [8–10].

Novel PrEP modalities, including long-acting vaginal rings, injectables, and implants, hold great promise for supporting uptake and overcoming barriers to daily oral PrEP adherence among PWID [7, 11, 12]. Long-acting injectable PrEP (LAI-PrEP) with cabotegravir injected every 8 weeks, recently approved by the United States’ Food and Drug Administration to prevent sexual HIV acquisition [13, 14], is hypothesized to be superior to daily oral tenofovir disoproxil fumarate/emtricitabine (TDF/FTC) as PrEP for preventing HIV acquisition via injection drug use [15, 16]. While few studies have assessed interest in and acceptability of novel long-acting PrEP modalities among PWID [11, 12, 17, 18], as many as half in some study samples have expressed interest in these formulations due to their perceived convenience of use, lack of invasiveness, and familiarity [11, 17, 18]. However, little is known about preferences for specific PrEP program characteristics (e.g., service location, prescription access location, adherence supports) among PWID, or the extent to which PrEP modality and programmatic characteristics considered together may drive PrEP engagement in this population.

Discrete choice experiment (DCE) methods are often used in market-based research to simulate consumers’ decision-making processes when faced with alternative products in the marketplace [19]. In a DCE, participants are presented with sets of product alternatives with varying characteristics and asked to choose between alternatives within each set. Participants’ choices are then modeled as a function of product characteristics to estimate the relative importance of product characteristics in driving consumers choices. A recent systematic review of DCEs engaging men who have sex with men (MSM) and transgender women (TGW) found that PrEP dosing frequency, effectiveness, and cost were the most important characteristics contributing to PrEP program interest [20]. To our knowledge, however, very little market-based research has been conducted with PWID. One of the few existing studies using market-based research methods (i.e., conjoint analysis, a related approach [19]), conducted with PWID in Ukraine, found that combined PrEP modality/frequency (e.g., daily pill, monthly injection) and dispensing site (e.g., pharmacy, family planning clinic) were key drivers of PrEP program interest [21]. No studies to our knowledge have examined preferences for PrEP modality or programmatic characteristics and their influences on PrEP program interest among PWID in North America [21], and none have explicitly investigated preferences for adherence supports (e.g., social support, street outreach), which could help increase overall PrEP interest and engagement for this population [22]. To inform interventions supporting engagement with PrEP, we conducted a DCE to assess preferences surrounding PrEP modality, frequency of use, service delivery and prescription access locations, and adherence supports, and the influence of these characteristics on willingness to engage with various PrEP programs among PWID.

## Methods

### Study population

HIV transmission across the San Diego-Tijuana border is ongoing [23, 24], and predictions suggest that injection drug use may account for nearly 50% of HIV infections in Tijuana by 2029 [25], highlighting an urgent need for prevention interventions, including PrEP, that are tailored to the needs and preferences of PWID in this region. Therefore, between May 2022 and June 2023, we conducted a DCE among PWID in San Diego County leveraging the infrastructure of two ongoing, interrelated studies in the San Diego-Tijuana border region. These two studies included (1) the “*La Frontera”* cohort study [26], and (2) the embedded “*LinkUP”* COVID-19 testing and vaccination uptake intervention pilot trial (in which some participants were also enrolled in *La Frontera*) [27]. Eligibility for both studies included being at least 18 years of age and reporting injection drug use in the past month. Additional eligibility criteria for *LinkUP* related to COVID-19 testing and vaccination history, as previously described [27], and agreeing to be recontacted for future research. For *La Frontera* participants included in this DCE (n = 179, 75% of those offered participation), DCE assessments were administered at either their 3- or 9-month locator check-in visits, where each participant completed only one DCE assessment. For *LinkUP* participants included in this DCE (n = 97, 91% of those offered participation), DCE assessments were administered within one week of their baseline assessment completion. Participants received $15 as compensation for their time completing the DCE assessment. All participants provided written informed consent, and all study procedures were approved by Institutional Review Boards at the University of California, San Diego, and Xochicalco University in Tijuana.

### Data collection

Surveys were interviewer-administered in English or Spanish, depending on participants’ preferences. *La Frontera* and *LinkUP* baseline surveys collected data on socio-demographics, injection and substance use behaviors, sexual behaviors, and PrEP-related variables. *Socio-demographics* included a participant’s age, sex assigned at birth (male or female), gender identity (man, woman, transwoman, transman, nonbinary, and other), ethnicity (Hispanic or non-Hispanic), highest level of education (none, incomplete/complete: primary, secondary, preparatory, and technical/trade, university/college, and other), and housing status (assessed through the questions, “In the past 6 months, tell me if you have lived in or slept in any of the following places,” and “In which place did you sleep in most of the time?”, with responses dichotomized into “unhoused” [shelter, welfare residence, workplace, car, bus, truck or other vehicle, abandoned building, migrant worker camp, asylum seeker shelter, deportee shelter, street, beach, canal, shooting gallery] and “housed” [own house or apartment, house or apartment of relatives/partners/friends, hotel/rented room, correctional institution, drug treatment center, medical care facility]). *Injection and substance use behaviors* in the past six months included fentanyl injection, polydrug use (using any two of the following: heroin, crack cocaine, fentanyl, ecstasy, methamphetamine, including simultaneous injection of these substances), injection frequency (dichotomized as multiple times daily vs. once daily or less), and receptive syringe use. *Sexual behaviors* in the past six months included sexual intercourse (vaginal, anal, or oral sex), transactional sex (exchanging money, drugs, alcohol, shelter, food, transportation, or other goods/services), alcohol or drug use before or during sex, and number of sexual partners. *PrEP-related variables* included prior PrEP awareness (assessed using the question, “Before today, had you ever heard of HIV-negative people taking HIV medications or PrEP *before* being exposed to HIV to protect against HIV infection?”) and prior PrEP use (assessed using the question, “Have you ever taken HIV medications or PrEP *before* being exposed to HIV to protect against HIV infection?”).

**Fig. 1:**
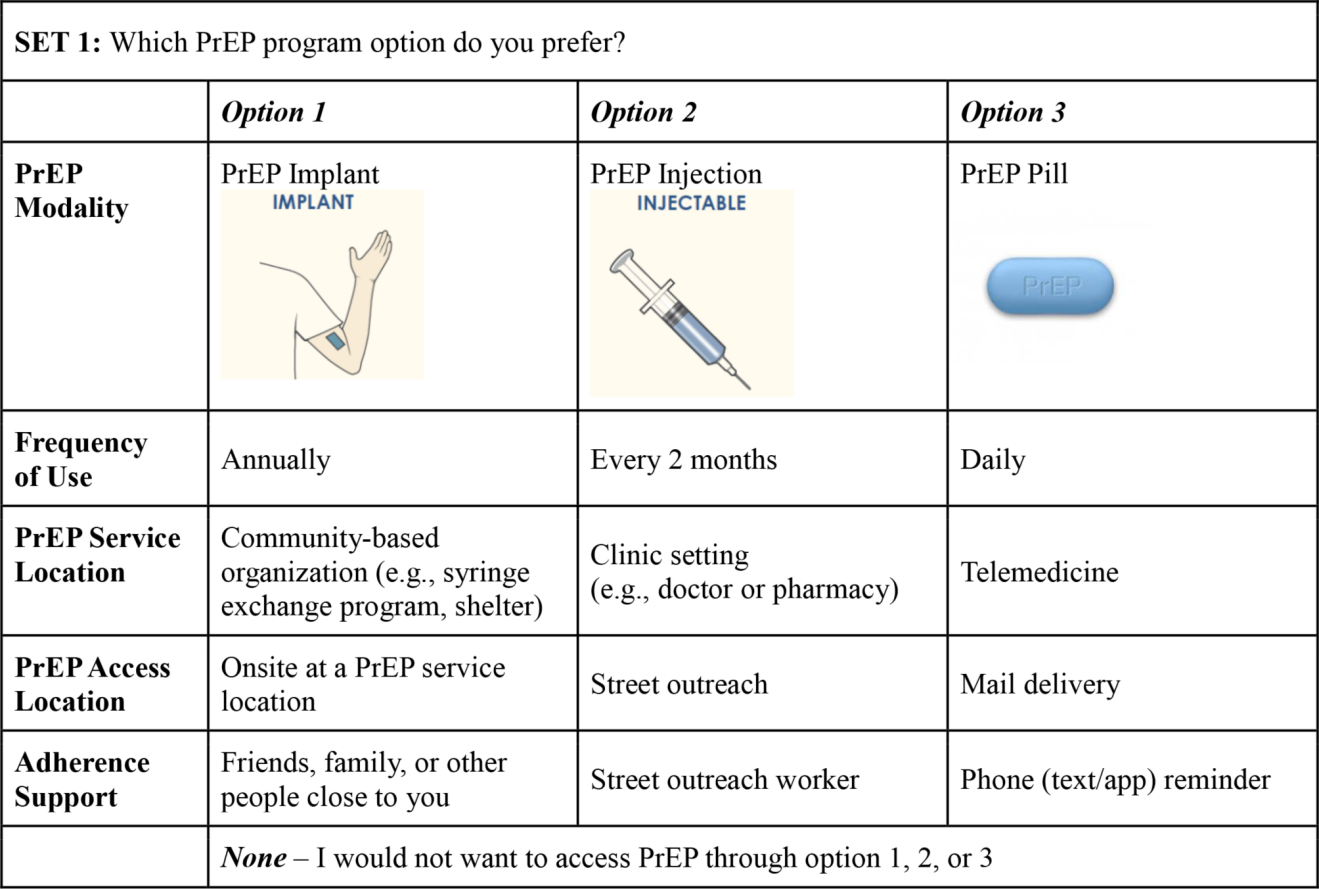
Example of DCE programmatic scenario task [47]. *Note* To inform participant’s choice of potentially unfamiliar products, interviewers showed pictures and descriptions of options before moving forward with the DCE. Photos are adapted from the National Institute of Allergy and Infectious Diseases (NIAID) “Long-Acting Forms of HIV Prevention” infographic [47]. *Abbreviations* DCE = Discrete Choice Experiment; PrEP = HIV pre-exposure prophylaxis

**Fig. 2:**
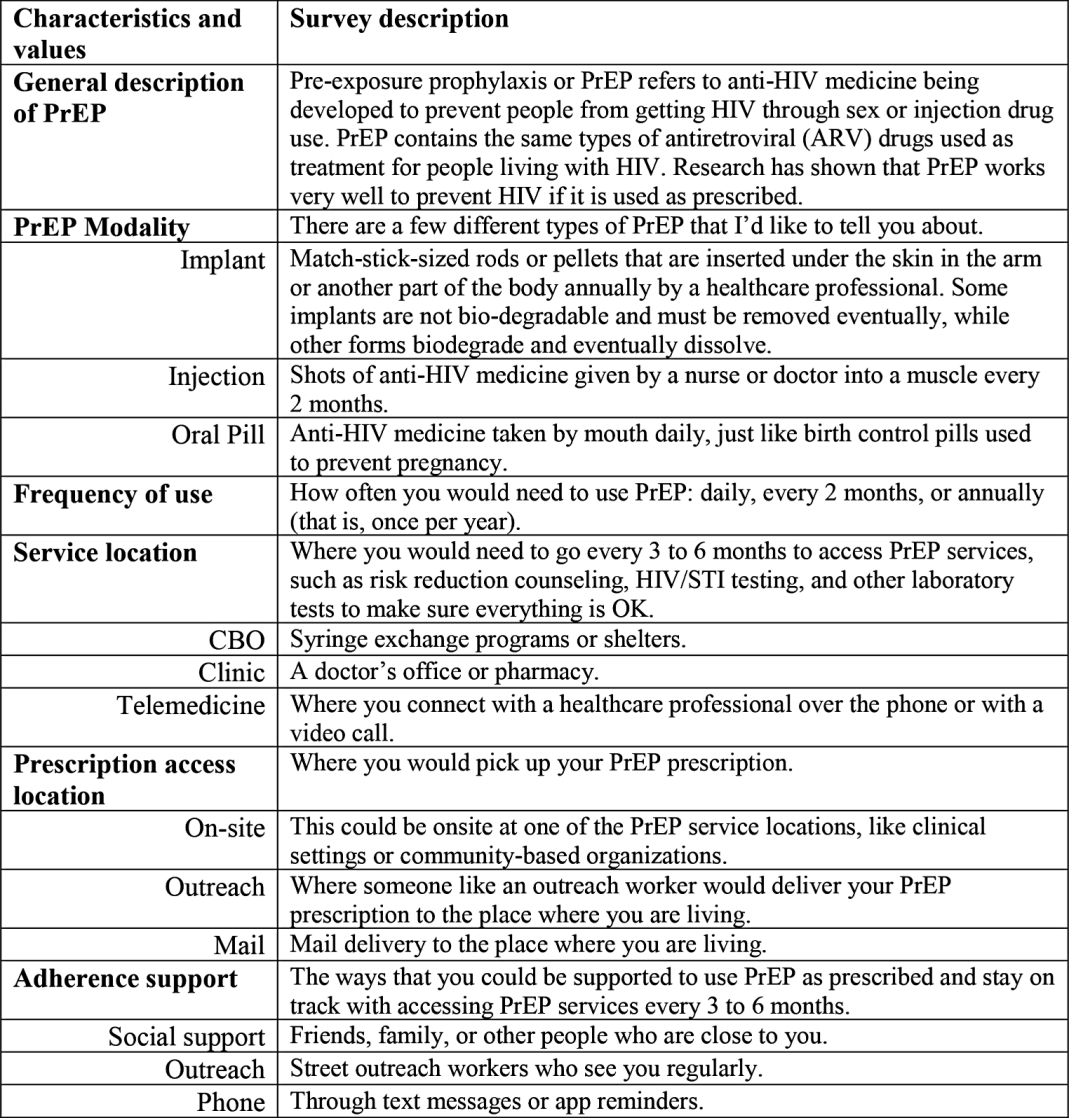
Description of PrEP modality and programmatic characteristics and associated values presented to participants. *Note* All descriptions were also provided in Spanish, which was validated by bilingual members of the research team. *Abbreviations* PrEP = HIV pre-exposure prophylaxis; CBO = community-based organization

We developed DCE surveys using specialized algorithms to select an efficient, balanced, and orthogonal experimental design [28], which included 18 different PrEP program scenarios that were presented to participants in sets of three. Participants were then asked to choose their preferred scenario within each set, where each set also included a “None” option that participants could choose if they had no interest in accessing PrEP via one of the three scenarios in each set (Fig. 1). Based on prior PrEP acceptability and DCE studies [20, 29–34], PrEP program scenarios consisted of various combinations of the following characteristics: *PrEP modality* (injectable, implantable, oral), *frequency of use* (annual, bi-monthly, daily), *service location* (community-based organization, clinic, telemedicine), *prescription access location* (on-site, street outreach, mail), and *adherence supports* (social support, outreach worker, phone/text reminder). Before presenting PrEP program scenarios to participants, interviewers briefly introduced PrEP and the PrEP program characteristics with examples to facilitate comprehension (Fig. 2).

### Statistical analysis

We used multinomial logit regression to model the probability of choosing PrEP program scenarios as a function of the five PrEP program characteristics (i.e., PrEP modality, frequency of use, service location, prescription access location, and adherence support) to estimate part-worth utility scores (PWUS) and relative importance scores (RIS) [28]. We averaged regression coefficients for each characteristic and then subtracted the average score from each score to calculate zero-centered PWUS, which sum to zero and reflect relative preferences for specific PrEP program characteristic values. Positive PWUS for a characteristic’s value indicate a relative preference for that value. Negative PWUS for a characteristic’s value indicate a relative aversion to that value. We calculated RIS for each characteristic by dividing the range of PWUS for a characteristic by the sum of the absolute value for the PWUS ranges multiplied by 100 [35, 36]. RIS reflect the relative influence of each characteristic on choice. Characteristics with higher RIS have a greater influence on participants’ PrEP program choices. To explore whether PWUS and RIS varied by factors we hypothesized could modify PrEP programmatic preferences, we also fit models stratified by sex assigned at birth, housing status, injection frequency, and prior PrEP awareness. For purposes of this analysis, we excluded 14 participants (6 from *LinkUp* and 8 from *La Frontera*) who self-reported testing positive for HIV, for a total sample size of 262. We conducted all analyses using SAS 9.4 (SAS Institute, Inc.; Cary, NC).

## Results

### Sample characteristics

Among 262 HIV-negative participants, mean age was 43.1 years (standard deviation [SD]: 11.8), 30.5% were assigned female sex at birth and identified as women, 92.0% identified as heterosexual/straight, 39.7% identified as Hispanic, 39.3% had completed secondary school or below, and 29.8% were unhoused (Table 1). Many participants reported injection and substance use behaviors known to increase the risk of HIV transmission, including past 6-month fentanyl injection (33.7%), polydrug use (75.9%), injecting multiple times daily (61.8%), and receptive syringe use (35.9%). A little over half reported having any sex (58.9%) and using alcohol or drugs before or during sex (55.0%), while very few reported transactional sex in the past 6 months (5.4%). Most participants were previously unaware of PrEP (75.2%), and only five (2.6%) reported ever using it.

**Table 1:** Characteristics of PWID in San Diego County (N = 262)

Characteristic	Total (N = 262) N (%)^a^
**Sociodemographics**	
**Mean age in years (SD)**	43.11 (11.8)
**Sex assigned at birth**	
Male	182 (69.5)
Female	80 (30.5)
**Gender identity**	
Man	182 (69.5)
Woman	80 (30.5)
**Sexual orientation**	
Heterosexual/Straight	241 (92.0)
Homosexual/Gay/Lesbian	7 (2.7)
Bisexual	13 (5.0)
Other	1 (0.4)
**Ethnicity**	
Hispanic	104 (39.7)
Non-Hispanic	158 (60.3)
**Highest level of education completed**	
Completed secondary and below	103 (39.3)
Some education beyond secondary	159 (60.7)
**Housing status (past 6 months)**	
Unhoused	184 (70.2)
Housed	78 (29.8)
**Injection and substance use behaviors**	
**Fentanyl injection (past 6 months)**	
Injected fentanyl	88 (33.7)
Did not inject fentanyl	173 (66.3)
**Polydrug use (past 6 months)** ^**b**^	
Polydrug use	198 (75.9)
No polydrug use	63 (24.1)
**Injection frequency (past 6 months)**	
Injected multiple times daily	162 (61.8)
Did not inject multiple times daily	100 (38.2)
**Receptive syringe use (past 6 months)**	
Injected using previously used syringe	94 (35.9)
Did not inject with a previously used syringe	168 (64.1)
**Sexual behaviors**	
**Any sex (past 6 months)** ^**c**^	
Had sex	153 (58.9)
Did not have sex	107 (41.2)
**Mean number of sexual partners (SD)**	1.32 (2.8)
**Transactional sex (past 6 months)** ^**d**^	
Transactional sex	14 (5.4)
No transactional sex	246 (94.6)
**Substance use before or during sex (past 6 months)** ^**e**^	
Used substances before or during sex	143 (55.0)
Did not use substances before or during sex	117 (45.0)
**PrEP related variables**	
**Prior PrEP awareness**	
Not aware of PrEP	197 (75.2)
Aware of PrEP	65 (24.8)
**PrEP use (ever)**	
Never used PrEP	191 (97.5)
Ever used PrEP	5 (2.6)

^a^ Percentage (%) values may not add up to 100% due to rounding; Ns may not sum to column total due to missing values

^b^ Defined as using any two of the following in the past 6 months: heroin, crack cocaine, fentanyl, ecstasy, PCP/Angel Dust, and methamphetamine; including simultaneous injection of crack and heroin, methamphetamine and crack, methamphetamine and heroin, and fentanyl and methamphetamine

Abbreviations: PWID = people who inject drugs; SD = standard deviation; PrEP = HIV pre-exposure prophylaxis

### Relative importance and part-worth utility scores

Frequency of use (RIS: 51.47) and PrEP modality (RIS: 35.26) had the greatest influence on participants’ PrEP program choices, while service location (RIS: 5.00), prescription access location (RIS: 3.20), and adherence supports (RIS: 5.07) were less influential (Table 2). The influence of these characteristics on participants’ PrEP program choices (i.e., RIS) did not differ meaningfully by sex assigned at birth, housing status, or injection frequency (data not shown). However, prescription access location (RIS: 8.98) and adherence supports (RIS: 10.08) had a slightly greater influence on PrEP program choices among participants with prior PrEP awareness (Table 2).

**Table 2:** RIS^a^ for PrEP programmatic characteristics, stratified by prior PrEP awareness

PrEP program characteristics	Total sample (N = 262)	Not Aware of PrEP (n = 197)	Aware of PrEP (n = 65)
Modality	35.26	36.10	36.61
Frequency of use	51.47	52.19	43.02
Service location	5.00	4.47	5.31
Prescription access location	3.20	2.00	8.98
Adherence support	5.07	5.24	10.08

^a^ RIS = relative importance scores, which reflect the influence of each characteristic on PrEP program decision-making (standardized to sum 100)

*Abbreviations* PrEP = HIV pre-exposure prophylaxis; RIS = Relative importance score

With respect to frequency of use, participants had a strong relative preference for annual PrEP use (PWUS: 0.834) and a relatively strong aversion to daily PrEP use (PWUS: -0.760) (Table 3). With respect to modality, participants preferred oral PrEP (PWUS: 0.566) and had an aversion to implantable PrEP (PWUS: -0.526). Participants did not have strong preferences for service or prescription access locations or adherence supports, as indicated by PWUS that were small in magnitude (i.e., close to zero). Although there were slight preferences for accessing PrEP via mail (PWUS: 0.222) and receiving adherence support by phone (PWUS: 0.192) among participants with prior PrEP awareness, there were no other meaningful differences in participants’ preferences for specific characteristic values (i.e., PWUS) by sex assigned at birth, housing status, or injection frequency (data not shown).

**Table 3:** Zero-centered^a^ part-worth utility scores (PWUS) for PrEP programmatic characteristics, stratified by prior PrEP awareness (N = 262)

PrEP program characteristics	Total Sample (N = 262)	Not Aware of PrEP (n = 197)	Aware of PrEP (n = 65)
**Modality**			
Implant	-0.526	-0.537	-0.802
Injection	-0.040	-0.007	0.013
Oral	0.566	0.545	0.789
**Frequency of use**			
Annually	0.834	0.856	0.923
Every two months	-0.074	-0.148	0.253
Daily	-0.760	-0.708	-1.176
**Service location**			
CBO	-0.058	-0.044	-0.136
Clinic	0.097	0.089	0.123
Telemedicine	-0.038	-0.045	0.013
**Prescription access location**			
On-site	-0.063	-0.039	-0.216
Outreach	0.036	0.018	-0.006
Mail	0.027	0.021	0.222
**Adherence support**			
Social support	-0.031	-0.035	0.108
Outreach	0.095	-0.061	-0.300
Phone	0.126	0.096	0.192
**Sum of ranges**	3.097	2.997	4.879

^a^ PWUS = zero-centered part-worth utility scores, which sum to zero and reflect relative preferences for specific values of a given PrEP program characteristic

*Abbreviations* CBO = Community-based organization; PrEP = HIV pre-exposure prophylaxis; PWUS = Part-worth utility scores

## Discussion

This is the first discrete choice experiment (DCE), to our knowledge, to thoroughly examine preferences for specific PrEP modalities and programmatic characteristics among PWID in North America. In our sample from the San Diego-Tijuana border region, participant preferences suggested that PrEP formulated as a long-acting pill may increase engagement with PrEP among PWID. Though development of long-acting PrEP pills has been attempted with some success in preclinical studies [37], clinical trials in humans were halted due to safety concerns [38]. Here, we highlight the potential importance of renewed investment in long-acting oral formulations to advance PrEP uptake for PWID.

In our study, frequency of PrEP use emerged as the most influential characteristic related to potential PrEP program engagement, with limited frequency of use requirements (e.g., annual use) being the most preferred. Our findings align with those from a previous study conducted among PWID in Ukraine, which revealed that more than 50% of PrEP program decision-making was influenced by PrEP modality/frequency of use [21]. However, unlike our assessment, previous research has conflated frequency of use and modality, by combining these attributes (i.e., modality and frequency: “oral PrEP taken daily”) [21]. By considering these attributes separately in our study, we found that PWID may most strongly prefer PrEP formulated as a long-acting (i.e., annual) pill, a hypothetical product that could increase PrEP interest, motivation, and uptake in this population if developed and made available [38]. Though more research on preferences for hypothetical PrEP products is needed, this interpretation is supported by findings from the Ukraine study, in which the second most preferred PrEP program characteristic was limited frequency of HIV testing [21], likely reflecting desires for the least burdensome PrEP “requirements” possible. Considering the significant competing needs and multilevel vulnerabilities experienced among PWID that limit healthcare service utilization (e.g., housing instability, limited transportation) [22, 39, 40], PrEP products and programs that minimize the frequency of use and other requirements (i.e., HIV testing) could help increase overall interest and engagement with PrEP among PWID.

PrEP modality also greatly influenced participants’ decision-making when choosing between alternative PrEP programs. Participants in our sample preferred oral PrEP, had no preferences for or aversions to injectable PrEP, and had stronger aversions to implantable PrEP. Prior qualitative evidence suggests that PWID prefer PrEP options that are convenient, non-invasive, and familiar (i.e., based on experience) [11], which may help explain why our participants preferred oral PrEP over other modalities. Indeed, oral PrEP circumvents the need for frequent clinic visits, procedural requirements, and is familiar due to the widespread prevalence of oral pills relative to injectables and implants [38]. However, the preferences for oral PrEP observed here differ from existing literature finding that PWID may prefer long-acting injectable cabotegravir injected every 8 weeks [6, 11, 18]. As noted above, however, participants in our study were able to pair their preferred modality with their desired frequency of use in hypothetical scenarios where they preferred longer-acting oral formulations. As such, future research focused on the development of long-acting oral PrEP formulations that are less invasive than injections may better serve the needs of PWID.

Although PrEP service and access locations and adherence supports appeared to have little influence on most participants’ PrEP program decision-making in our study, those with prior PrEP awareness expressed some slight preferences related to these attributes. Specifically, these participants preferred mail delivery of PrEP prescriptions and receiving adherence support by phone. These preferences align with the overarching theme that PWID are inclined towards PrEP alternatives that reduce the burdens or “requirements” of PrEP-related care, such as in-person healthcare appointments that may be difficult for them to access [6, 11, 21, 41]. PWID often contend with stigma in healthcare settings [42–45], which may result in a desire to access PrEP in alternative formats. If given remote PrEP prescription access (i.e., mail) and adherence reminder (i.e., phone) options, which are not yet widely available to PWID, this population might be more motivated and successful in engaging with PrEP programs.

Our findings should be interpreted within the context of several limitations. First, this study was conducted with PWID already engaged in ongoing research studies in the San Diego-Tijuana border region, a unique setting that may limit the generalizability of our findings. Second, we were unable to detect differences in the influence of or preferences for PrEP programmatic characteristics by sex assigned at birth, housing status, and injection frequency; larger samples may be required for more robust research on these and other hypothesized modifiers of PrEP-related preferences. Third, our use of self-reported behavioral measures may have introduced potential response bias and recall errors. Fourth, to minimize the burden on study participants, our DCE focused on five key PrEP programmatic characteristics and did not explore some characteristics (e.g., cost) that could also influence PrEP program engagement in this population [29]. Finally, actual PrEP program engagement will likely differ from the hypothetical PrEP program choices made available in our DCE. While 8% (*n* = 22) of participants selected the “None” option in response to at least one set of PrEP program scenarios presented to them, only 3% (*n* = 9) of participants selected the “None” option across all of the PrEP program scenario sets, suggesting that our findings may reflect the PrEP program preferences of PWID with a greater likelihood of engaging in PrEP care. Addressing these limitations through future research could help contribute to a more comprehensive understanding of PrEP preferences and their implications for advancing PrEP engagement among PWID.

## Conclusion

Findings from this discrete choice experiment (DCE) provide important insights into the characteristics influencing PrEP program choices among people who inject drugs (PWID). This study highlights the significance of frequency of use and PrEP modality in shaping PrEP program preferences among PWID, with annual use and oral PrEP being the most preferred options. To enhance PrEP uptake and adherence among PWID, additional efforts are needed to engage this population in product development and testing research while continuing to explore intervention and implementation strategies that can engage and retain PWID in this expanding set of biomedical HIV prevention tools [46].

## Data Availability

Data are available upon reasonable request to the Principal Investigators of *La Frontera*, Dr. Steffanie Strathdee (sstrathdee@health.ucsd.edu), and *LinkUp*, Dr. Angela R. Bazzi (abazzi@health.ucsd.edu).

## Abbreviations

CBO: Community based organization
DCE: Discrete choice experiment
LAI-PrEP: Long-acting injectable pre-exposure prophylaxis
MSM: Men who have sex with men
PrEP: Pre-exposure prophylaxis
PWID: People who inject drugs
PWUS: Part-worth utility scores
RIS: Relative importance scores
TDF/FTC: Tenofovir disoproxil fumarate/emtricitabine
TGW: Transgender women
SD: Standard deviation
